# Substance-Specific and Shared Gray Matter Signatures in Alcohol, Opioid, and Polysubstance Use Disorder

**DOI:** 10.3389/fpsyt.2021.795299

**Published:** 2022-01-18

**Authors:** Angela M. Muller, David L. Pennington, Dieter J. Meyerhoff

**Affiliations:** ^1^Department of Radiology and Biomedical Imaging, University of California, San Francisco, San Francisco, CA, United States; ^2^VA Advanced Imaging Research Center (VAARC), San Francisco VA Medical Center, San Francisco, CA, United States; ^3^Department of Psychiatry and Behavioral Sciences, University of California, San Francisco, San Francisco, CA, United States; ^4^San Francisco Veterans Affairs Health Care System (SFVAHCS), San Francisco, CA, United States

**Keywords:** polysubstance use disorder, cortical thickness, gray matter volume, frontocerebellar circuit, anterior insula, medial superior frontal gyrus

## Abstract

Substance use disorders (SUD) have been shown to be associated with gray matter (GM) loss, particularly in the frontal cortex. However, unclear is to what degree these regional GM alterations are substance-specific or shared across different substances, and if these regional GM alterations are independent of each other or the result of system-level processes at the intrinsic connectivity network level. The T1 weighted MRI data of 65 treated patients with alcohol use disorder (AUD), 27 patients with opioid use disorder (OUD) on maintenance therapy, 21 treated patients with stimulant use disorder comorbid with alcohol use disorder (polysubstance use disorder patients, PSU), and 21 healthy controls were examined via data-driven vertex-wise and voxel-wise GM analyses. Then, structural covariance analyses and open-access fMRI database analyses were used to map the cortical thinning patterns found in the three SUD groups onto intrinsic functional systems. Among AUD and OUD, we identified both common cortical thinning in right anterior brain regions as well as SUD-specific regional GM alterations that were not present in the PSU group. Furthermore, AUD patients had not only the most extended regional thinning but also significantly smaller subcortical structures and cerebellum relative to controls, OUD and PSU individuals. The system-level analyses revealed that AUD and OUD showed cortical thinning in several functional systems. In the AUD group the default mode network was clearly most affected, followed by the salience and executive control networks, whereas the salience and somatomotor network were highlighted as critical for understanding OUD. Structural brain alterations in groups with different SUDs are largely unique in their spatial extent and functional network correlates.

## Introduction

Moderate to severe substance use disorder (SUD) is routinely associated with gray matter (GM) alterations, usually GM loss, in SUD individuals relative to controls (1–3) and with impairments in cognition and mood (4–7). However, because the studies investigating GM alterations in SUD usually focus on one specific SUD at a time, the question of regional specificity of GM changes for different substances or of whether there is a GM correlate common to all SUDs is still largely unanswered. A recent mega-analysis (2) compared cortical thickness and subcortical GM volume in 1,100 healthy controls and 2,140 SUD individuals using one of five different substances (alcohol, nicotine, cocaine, methamphetamine, or cannabis). Compared to controls, seven brain regions consisting of bilateral insula and middle temporal gyrus, left inferior parietal cortex, and supramarginal gyrus as well as right medial orbitofrontal cortex showed cortical thinning across all five SUDs; a common subcortical structure with GM volume loss, however, could not be identified (2). Further, only individuals with alcohol use disorder (AUD) and cocaine use disorder showed substance-specific cortical thinning but not individuals with nicotine, methamphetamine, or cannabis use disorder (2). In a follow-up study with a somewhat larger sample of 2,277 SUD individuals and 1,628 controls, the same authors used more fine-grained morphological shape analyses of subcortical GM structures to identify substance-specific and substance-general alterations in the same five SUD subgroups (1). In comparison to non-dependent controls, AUD was associated with smaller hippocampus, thalamus, putamen, and amygdala volumes surfaces and thicknesses, whereas participants with nicotine use disorder showed greater volumes in bilateral hippocampus and right nucleus accumbens. Interestingly, the authors did not find any subcortical shape alterations unique to the other three investigated SUD subgroups (1).

These two mega-analyses aimed to identify distinct and unique GM features vs. commonly shared features in monosubstance use disorders, but 11.3% of treatment seeking SUD individuals use at least alcohol and an illegal substance concurrently, i.e., use polysubstances, and their prevalence in treatment centers is consistently increasing (8, 9). Only a small number of studies have attempted to determine the effects of polysubstance use disorder (PSU) on brain tissue. Grodin et al. (10) using a data-driven voxel-based morphometry (VBM) approach observed significant GM differences in mesial frontal lobe and right temporal lobe in AUD and alcohol abusing PSU individuals combined when compared to controls, and subcortical changes similar to those seen in Wernicke-Korsakoff Syndrome in the AUD individuals when compared to the PSU group. In a follow-up study focusing on the subcortical structures in AUD and PSU, Grodin et al. (11) reported that PSU individuals had only reduced volume in the bilateral thalamus, whereas AUD individuals had volume loss in bilateral hippocampus, right nucleus accumbens, and thalamus when compared to controls. When compared to each other, PSU had larger right caudate volume than the AUD group. We previously described (12) that AUD individuals consistently had smaller normalized white matter (WM) volume than PSU across all major brain lobes, and PSU had even larger frontal and parietal WM volumes than controls, but smaller temporal GM volumes, and smaller putamen, globus pallidus and thalamus volumes than controls. Those differences were observed despite similar lifetime histories of alcohol consumption in the two groups. Using a region of interest (ROI) approach with a priori defined frontal brain regions, Pennington et al. (6) reported that PSU individuals had significantly smaller left orbitofrontal GM volume and surface area than controls, and a significantly thinner right anterior cingulate gyrus than AUD individuals. Thus, comorbid SUD in AUD patients does not simply amplify structural deficits specific to AUD.

Determining unique regional substance-specific GM alterations or even identifying regional GM alterations shared by several SUDs does not answer the questions whether these regional GM alterations are independent from each or whether they related to each other insofar as they are the result of system-level processes. Motivated by the observation that the GM alterations patterns in several neurodegenerative syndromes were strikingly similar to the spatial distribution of resting-state networks or intrinsic connectivity networks (ICN), Seeley et al. (13) tested the hypothesis that typical GM alterations reliably found in five sub-types of dementia rather reflect a syndrome-specific system-level process than several independent regional effects; they used a combination of VBM, structural covariance analysis, and seed correlation analyses (SCA) in resting-state fMRI data. Based on the high spatial similarity between the VBM patterns found in the five patient groups with the GM covariation patterns and SCA matrices, the authors (13) concluded that the five neurodegenerative syndromes did not evolve randomly and affect brain areas independently from each other, but that they targeted syndrome-specific networks closely resembling the system-level ICNs also found in healthy participants. Since the report of Seeley et al. (13), GM covariance analysis has not just been used to better understand the spreading of neurodegenerative disorders but also individual brain development over the lifespan (14–17), neurological conditions such as autism (18, 19), ADHD (20), traumatic brain injury (21), epilepsy (22), psychiatric disorders such as schizophrenia (23) and major depression (24). Furthermore, several meta-analyses including thousands of healthy control data sets have confirmed that the structural GM organization is recapitulating on a system-level the intrinsic functional organization of the brain with a high concordance of 64% (25) to 68% (26). These developments highlight the importance of system-level approaches to better understand the neuropathological processes in many other neurologic and neuropsychiatric disorders (26–29).

The purpose of our study was thus 2-fold: One, to determine vertex- and voxel-wise GM alterations across the entire brain unique to and shared by three common SUD groups: individuals with AUD, opioid use disorder (OUD), and stimulant use disorder comorbid with AUD (i.e., PSU); and two, to investigate by a combination of analysis methods used previously (13) if any of the observed regional GM alterations could be explained by intrinsic system-level processes.

## Methods

### Participants

T1 weighted MRI data from 21 PSU (2 African-American, 1 Asian, 12 Caucasian, 3 Latino, 3 Others), 65 AUD (9 African-American, 44 Caucasian, 5 Latino, 7 Others) and 27 OUD (6 African-American, 16 Caucasian, 1 Latino, 4 Others) individuals as well as 21 healthy non-drinking/light drinking controls (3 African-American, 5 Asian, 5 Caucasian, 2 Latino, 6 Others) were used for this study. The SUD individuals were recruited from outpatient treatment clinics in San Francisco, CA, controls were recruited from the local community. Some of the T1 weighted data of this study have been used for two previous publications (30, 31). PSU and AUD participants were ~1 month abstinent when scanned, OUD individuals were on maintenance medication when scanned. Group demographics and relevant clinical data are summarized by group in Table 1.

**Table 1:** Demographics by group, *t*-tests for group comparisons.

	**Sample size**	**Age**	**Education (Years)**	**Sex (Female/Male)**
CON	21	45.3 (8.3)	16.2 (2.1)	7/14
PSU	21	43.7 (11.1)	15.3 (2.3)^C^	4/17
AUD	65	41.8 (9.5)	14.8 (2.1)^A, D^	25/40
OUD	27	45.6 (11.9)	12.9 (1.3)^A, C, D^	5/21
	**Smoking status** **(non/current/former)**	**FTND total**	**Pack years** **(smokers only)**	
CON	14/2/5	3.25 (1.26)	3.9 (5.4)	
PSU	3/13/5^A, B^	3.78 (1.31)	13.0 (11.2)	
AUD	29/23/13^A, B, D^	3.62 (1.84)	11.8 (10.6)	
OUD	2/19/6^A, D^	3.96 (1.52)	14.8 (10.4)	
	**AUDIT**	**Lifetime average** **Drinks/Month**	**Onset year for heavy** alcohol drinking*	**Number of months of** heavy alcohol drinking*
CON	1.85 (1.18)	8.8 (7.1)		
PSU	26.56 (9.06)^A, C^	146.2 (137.8)^A^	23.6 (7.0)	185.1 (131.8)^C^
AUD	30.59 (6.49)^A, D^	188.8 (98.1)^A, D^	23.5 (7.8)	175.4 (97.1)^D^
OUD	8.04 (11.45)^A, C, D^	111.2 (151.7)^A, D^	21.2 (6.7)	70.9 (99.1)^C, D^
	**Beck depression** **inventory (Total)**	**STAI—State**	**STAI—Trait**	
CON	2.9 (3.6)	24.0 (5.2)	31.0 (8.7)	
PSU	14.1 (6.6)^A^	35.0 (11.9)^A^	47.7 (9.8)^A, C^	
AUD	13.8 (7.8)^A, D^	37.1 (12.1)^A, D^	47.4 (12.5)^A, D^	
OUD	10.2 (8.6)^A, D^	30.9 (8.7)^A, D^	36.1 (9.6)^C, D^	
	**Barrett impulsivity** **scale—total**	**Barrett impulsivity** **scale—attentional**	**Barrett impulsivity** **scale—motor**	**Barrett impulsivity** **scale—non-planning**
CON	53.7 (7.9)	13.0 (3.0)	19.9 (3.4)	20.8 (4.2)^A^
PSU	74.3 (8.2)^A, B, C^	19.8 (4.7)^A, C^	25.9 (3.8)^A, B^	28.5 (3.9)^A^
AUD	65.5 (11.4)^A, B^	18.0 (4.1)^A, D^	23.5 (4.6)^A, B^	25.8 (5.1)^A, B^
OUD	65.48 (10.1)^A, C^	15.6 (3.3)^A, C, D^	23.9 (4.4)^A^	26.0 (4.6)^A, B^

The table shows the mean values with the standard deviations in brackets; CON, controls; PSU, polysubstance use disorder individuals; AUD, alcohol use disorder individuals; OUD, opioid use disorder individual; FTND, Fagerström Test of Nicotine dependence; AUDIT, Alcohol Use Disorders Identification Test; STAI, State-Trait Anxiety Inventory. The letters indicate the following statistical comparisons and significance levels: A, statistical difference between controls vs. PSU, AUD, or OUD at p < 0.05; B, statistical difference between PSU vs. AUD at p < 0.05; C, statistical difference between PSU vs. OUD at p < 0.05; D, statistical difference between AUD vs. OUD at p < 0.05. *Heavy drinking was defined historically as consuming >100 alcoholic drinks per month before treatment.

The screening section of the Structural Clinical Interview for DSM-5 Axis I disorders was administered to all participants. All SUD individuals fulfilled the criteria for moderate or severe SUD with or without tobacco use disorder. AUD patients had consumed on average 292 standard alcoholic drinks (1 standard alcoholic drink contains 13 g of ethanol) per month for at least 6 years before treatment. PSU individuals had consumed on average 240 standard alcoholic drinks per month in combination with an average cocaine consumption of 11 grams per month before treatment (18 grams per month during the last year). OUD individuals had consumed on average 84 standard alcoholic drinks per month for at least 8 years and were in maintenance therapy for an average of 32 months when scanned, 18 of the 27 OUD individuals were taking an average of 45 mg methadone per day (SD = 40.6 mg) and the remaining 8 OUD patients took an average of 8.15 mg suboxone per day (SD = 10.8 mg). Controls had consumed on average of 7 standard alcoholic drinks in any month over lifetime and had not used any substances beyond recreational marijuana. Exclusion criteria for all participants included a history of neurologic disorder, e.g., epilepsy, traumatic brain injury with loss of consciences < 30 min, cerebrovascular disease, a history of general medical disease such as untreated hypertension, diabetes, hypo/hyperthyroidism, and of psychiatric diseases (e.g., major depression, anxiety, trauma, and PTSD).

All participants were assessed by a battery of in-person interviews and standardized questionnaires that included the Beck Depression Inventory [BDI, (32)], Barratt Impulsivity Scale [BIS 11, (33)], the State-Trait-Anxiety Inventory [STAI; (34)], the Alcohol Use Disorder Identification Test [AUDIT; (35)], the Fagerström Test for Nicotine Dependence test [FTND, (36)] as well as standardized questionnaires assessing lifetime substance use including tobacco. The Committees on Human Research at the University of California San Francisco and the San Francisco VA Health Care System had approved the study. Signed informed consent had been obtained from each participant prior to any research procedures in accordance with the Declaration of Helsinki.

### MRI Data

The MRI data were collected at the San Francisco VA Health Care System on a 3.0 T MRI scanner (Siemens Magnetom Skyra Syngo MR D13) using a 20 channel receive head coil. The study protocol included different types of structural images, as well as rs-fMRI data and spectroscopy data. For this study we used a T1 weighted MPRAGE sequence with repetition time (TR) = 2,300 ms; echo time (TE) = 2.98 ms; flip angle 90, field of view (FOV) 192 × 256 × 256 mm^3^, isotropic voxel size 1 × 1 × 1 mm^3^, 256 slices per volume, acquisition duration = 5.28 min.

### First Part—Morphological Analyses

#### Vertex-Wise Cortical Thickness (CT) Analyses

To examine differences in cross-sectional CT and subcortical volumes between the four groups, the Computational Anatomic Toolbox (CAT12 version 12.7) (http://www.neuro.uni-jena.de/cat/) was used, which is implemented in the Statistical Parametric Mapping Toolbox (SPM12) (http://www.fil.ion.ucl.ac.uk/spm/) and was run on MATLAB R2017b.

The processing pipeline of CAT12 for the computation of vertex-wise CT consists of three processing sub-procedures 1. The first sub-procedure starts with an “initial voxel-based processing” during which the T1 weighted image is denoised, resampled, initially bias-corrected, affine registered, and then segmented into the three tissue classes using the standard SPM Unified Segmentation procedure (37) 2. During the “refined voxel-based processing” sub-procedure, the output from the unified segmentation procedure is refined by skull-stripping of the brain, parcellation into left and right hemisphere, subcortical areas and cerebellum, local intensity correction. After this, the segmentation is further refined by a second segmentation step, which does not rely on a priori information of the tissue probabilities, and by applying a partial volume estimation. Finally, the tissue maps are spatially normalized to the MNI space using the Geodesic Shooting (38) registration 3. The “surface-based processing” sub-procedure relies on the input of the two VBM sub-procedures. To reconstruct the central surface and to estimate CT the projection-based thickness method is used (39). The topological defects are repaired followed by surface refinement resulting in the final central surface mesh. Then the individual central surfaces are registered to the Freesurfer “FsAverage” template and the local thickness values transferred to the Freesurfer “FsAverage” template. After this spatial registration the data were smoothed using a Gaussian filter of 12 mm.

#### Statistical Analyses CT

To test for group differences in vertex-wise CT, we used the SPM full factorial model with SUD status as factor and the four groups (CON, PSU, AUD, OUD) as levels, and age was defined as covariate without interest. Total intercranial volume (TIV) was not modeled as covariate without interest for the CT analyses, since cortical thickness does not linearly scale with brain size (40–42). For the same reason, neither sex nor education were modeled as covariates without interest (43, 44). As we were not interested to demonstrate a global difference in cortical thickness between healthy controls and SUD patients but to investigate whether specific cortical thickness differences exist between the four groups, six group contrasts were defined as *t*-tests: controls vs. PSU, controls vs. AUD, controls vs. OUD, PSU vs. AUD, PSU vs. OUD, AUD vs. OUD.

#### Statistical Analyses Subcortical and Cerebellar Volumes on Voxel-Level

To test for group-specific differences in GM volume in subcortical regions and cerebellum, the normalized GM probability maps from the VBM pipeline were smoothed with an 8 mm Gaussian filter. The same SPM full factorial model and the same statistical contrasts were used as for the CT analyses, but now age and TIV were modeled as covariates without interest. To constrain the VBM analysis on the subcortical regions and the cerebellum only, the corresponding ROIs (hippocampus, thalamus, caudate nucleus, putamen, globus pallidus, nucleus accumbens, and 18 cerebellum ROIs) from the AAL atlas (45) were used to build a binary mask, and the mask was implemented during the following non-parametric permutation procedure.

The non-parametric Threshold-Free-Cluster-Enhancement (TFCE; permutation with 10,000 iterations) method in combination with the FWE correction (threshold *p* ≤ 0.001 FWE) to control for multiple comparisons were used to detect vertex/voxel clusters indicating significant between-group differences in regional CT and subcortical-cerebellar GM volume. In contrast to other cluster-based thresholding methods, the TFCE method does not assume stationarity (= constant smoothness) of the data, provides better sensitivity as it is less affected by the smoothing kernel used, and does not require the user to arbitrarily specify an initial cluster-forming threshold (46, 47).

### Second Part—Mapping the Group-Specific Differences in CT to System-Level Functional Processes

First, to relate the vertex-wise CT reduction pattern in the three SUD subgroups to well-established functional systems, we repeated the CT analysis using a data-driven ROI approach at *p* = 0.01 FDR corrected in combination with the Schaefer parcellation (48). The parcellation comes in different resolutions (100, 200, 400, 600, and 600 parcels); the resolution with 200 parcels was used, because the results matched the spatial distribution of the vertex-wise analyses best. An additional feature of the Schaefer parcellation is that each parcel is assigned to one the 17 ICNs as identified by Yeo et al. (49). The 17 networks consist of a visual ICN, a temporoparietal ICN, a SMN (SMN) ICN with two subparts a and b, a limbic ICN with two subparts a and b, a dorsal attention (DAN) ICN with two subparts a and b, an executive control (ECN) ICN with three subparts a, b, and c, and a default mode (DMN) ICN with three subparts a, b, and c. A special feature of the Yeo et al. (49) network assignment is that the salience network (SAL) and the ventral attention network (VAN) are grouped together but further divided into subparts a and b.

In a next step, we computed the corresponding structural GM covariance patterns. To that purpose, the 200 parcels of the Schaefer parcellation were mapped on the unsmoothed, individual native space of each subject and then the mean CT value of each parcel was extracted. We then computed the mean CT value of each participant, subtracted that value from each of the participant's 200 CT ROI values and used the resulting CT values as dependent variables in a ROI-wise linear regression with the individual age of each participant as independent variable to control for potential age-confounds. The resulting residual CT values were used to compute a group-specific correlation matrix for each of the three SUD subgroups. Next, the two Schaefer ROIs were identified that contained the MNI coordinates of the two most significant peak vertices from the data-driven CT analysis, and then we determined for each of these seed ROIs separately with which of the other 199 ROIs it had a significant positive correlation (*p* < 0.05).

Last, following the rationale of Seeley et al. (13), the same MNI coordinates as from the step above were then used as input for a subsequent SCA with healthy participants on Neurosynth [(50); https://neurosynth.org/]. Neurosynth is a web-based platform for automatically synthesizing the results of a large number of different neuroimaging studies (14,371 studies, status August 2021), which also allows to compute a SCA for any MNI coordinate based on the resting-state data from 1,000 healthy participants.

## Results

### Demographics

Table 1 compares basic demographical data of the four groups studied. The groups did not differ in age and in the proportion of male vs. female participants. AUD and OUD had significantly fewer years of education than controls, and OUD had also significantly fewer years of education than PSU and AUD. The three SUD subgroups had a higher proportion of current and former smokers than controls, although the currently smoking participants of all four groups did not significantly differ in amount (pack years) or severity of smoking (FTND). All three SUD subgroups scored higher than controls in amount (lifetime average drinks per months) and severity (AUDIT) of alcohol drinking. OUDs drank less and had lower AUDIT scores than AUD and PSU individuals. As expected, the SUD subgroups had higher scores than controls for depression and state anxiety, but OUD had lower depression and trait anxiety symptomatology than AUD and PSU individuals. All SUD subgroups had higher BIS 11 impulsivity scores than controls, and PSU scored higher than both AUD and OUD on several of the subscores.

### First Part—GM Differences in the Four Groups

#### Group-Specific Differences in TIV, Global Atrophy, Global Tissue Volumes, and Mean Cortical Thickness

Table 2 shows that the four groups did not differ significantly in TIV and global GM volume, but AUD individuals had significantly less global WM volume and greater brain atrophy than controls, OUD and PSU. OUD individuals on average had a thinner GM ribbon than controls and the other two SUD subgroups.

**Table 2:** Total intracranial volume, global gray and white matter volume in cm^3^, and cortical thickness in mm.

	**Total intracranial brain volume**	**Gray matter volume (TIV corrected)**	**White matter volume (TIV corrected)**	**Atrophy (TIV corrected)**	**Average cortical thickness**
CON	1,493 (106)	429 (19)	357 (16)	1.27 (0.04)	2.52 (0.07)
PSU	1,568 (138)	427 (35)	364 (20)^B^	1.27 (0.07)^B^	2.49 (0.12)^C^
AUD	1,510 (141)	419 (28)	346 (21)^A, B, D^	1.31 (0.07)^A, B, D^	2.49 (0.08)^D^
OUD	1,522 (138)	423 (32)	363 (20)^D^	1.27 (0.06)^D^	2.44 (0.11)^A, C, D^

*The table shows the mean with the standard deviation in brackets; CON, controls; PSU, polysubstance use disorder individuals; AUD, alcohol use disorder individuals; OUD, opioid use disorder individuals; The letters indicate the following statistical comparisons and significance levels: A, statistical difference between controls vs. PSU, AUD, or OUD at p < 0.05; B, statistical difference between PSU vs. AUD at p < 0.05; C, statistical difference between PSU vs. OUD at p < 0.05; D, statistical difference between AUD vs. OUD at p < 0.05*.

#### Vertex-Wise CT Comparisons of the Three SUD Subgroups With Controls

**Figure 1:**
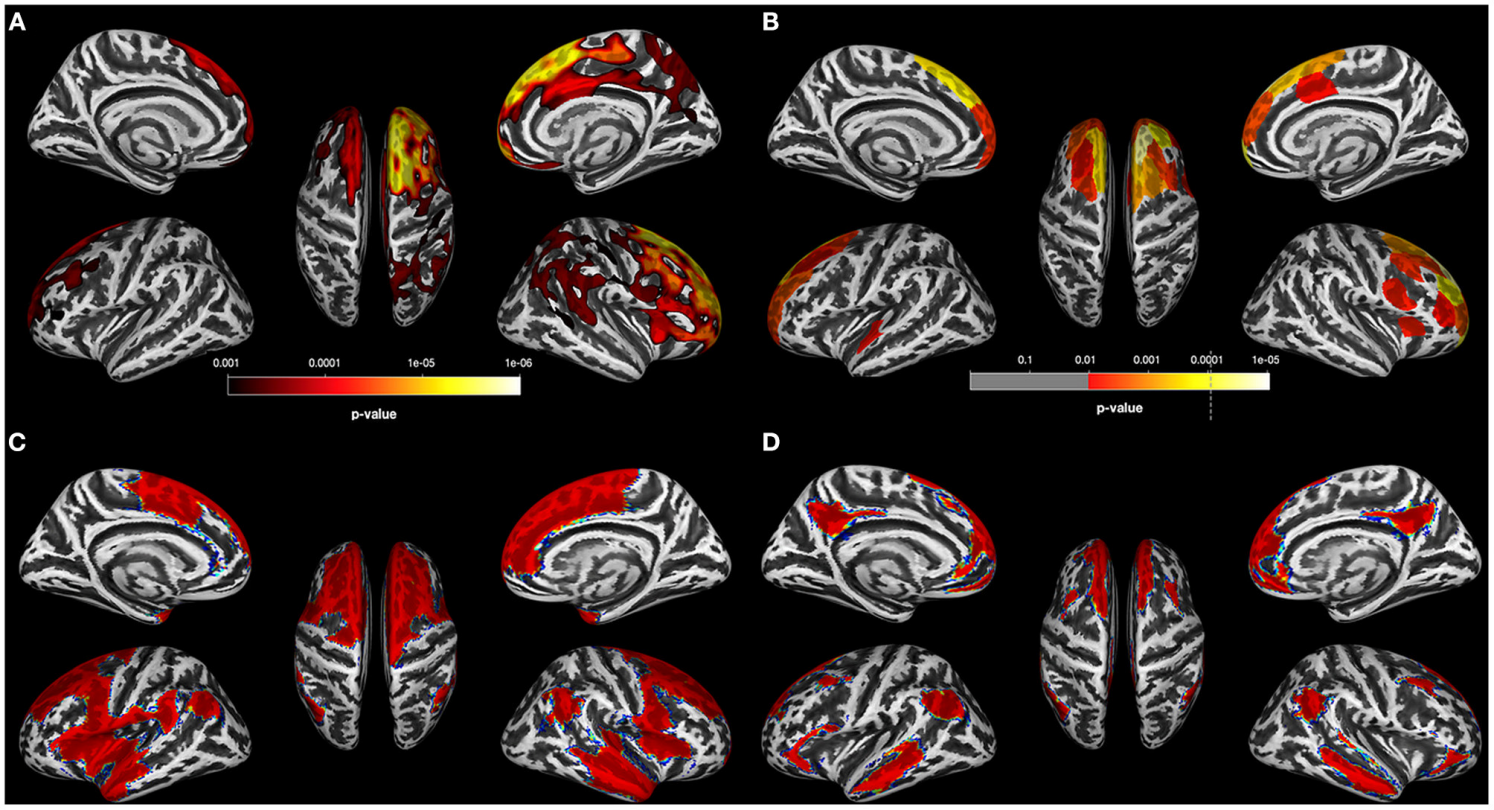
Results for AUD individuals **(A)**. Results of the data-driven vertex-wise cortical thickness (CT) analysis—controls have significantly higher CT values than alcohol use disorder individuals (AUD) at *p* = 0.001 FWE corr. (TFCE). **(B)** Results of the data-driven ROI analysis with the Schaefer Atlas (48) (200 parcels) colored are ROIs for which controls have significantly higher mean CT values than AUD at *p* = 0.01 FDR corr; **(C)** CT covariance results highlighted with red color all Schaefer ROIs with which the two ROIs corresponding to the two peak vertices of the most significant clusters in **(A)** show significant positive correlations at *p* < 0.05. **(D)** Result of the Neurosynth seed correlation analysis (thresholded at *r* > 0.2) with resting-state data of 1,000 healthy participants with the MNI coordinates of the two peak vertices of the most significant clusters in **(A)** as seeds.

When compared with controls, the AUD and OUD groups, but not the PSU group, showed reduced CT across large portions of the cortex at *p* = 0.001 FWE corr. (TFCE). In detail, AUD had thinner cortices in two large bilateral clusters with 59,928 vertices in the right hemisphere and 12,346 vertices in the left hemisphere. The right-hemispheric cluster had its peak vertex in the superior frontal gyrus and extended further into the middle and inferior frontal gyri, posterior part of the superior and middle temporal gyri, temporoparietal junction, and parietal cortex (Figure 1A). The smaller left-sided cluster had its peak vertex also in the superior frontal gyrus and extended into the middle frontal gyrus.

**Figure 2:**
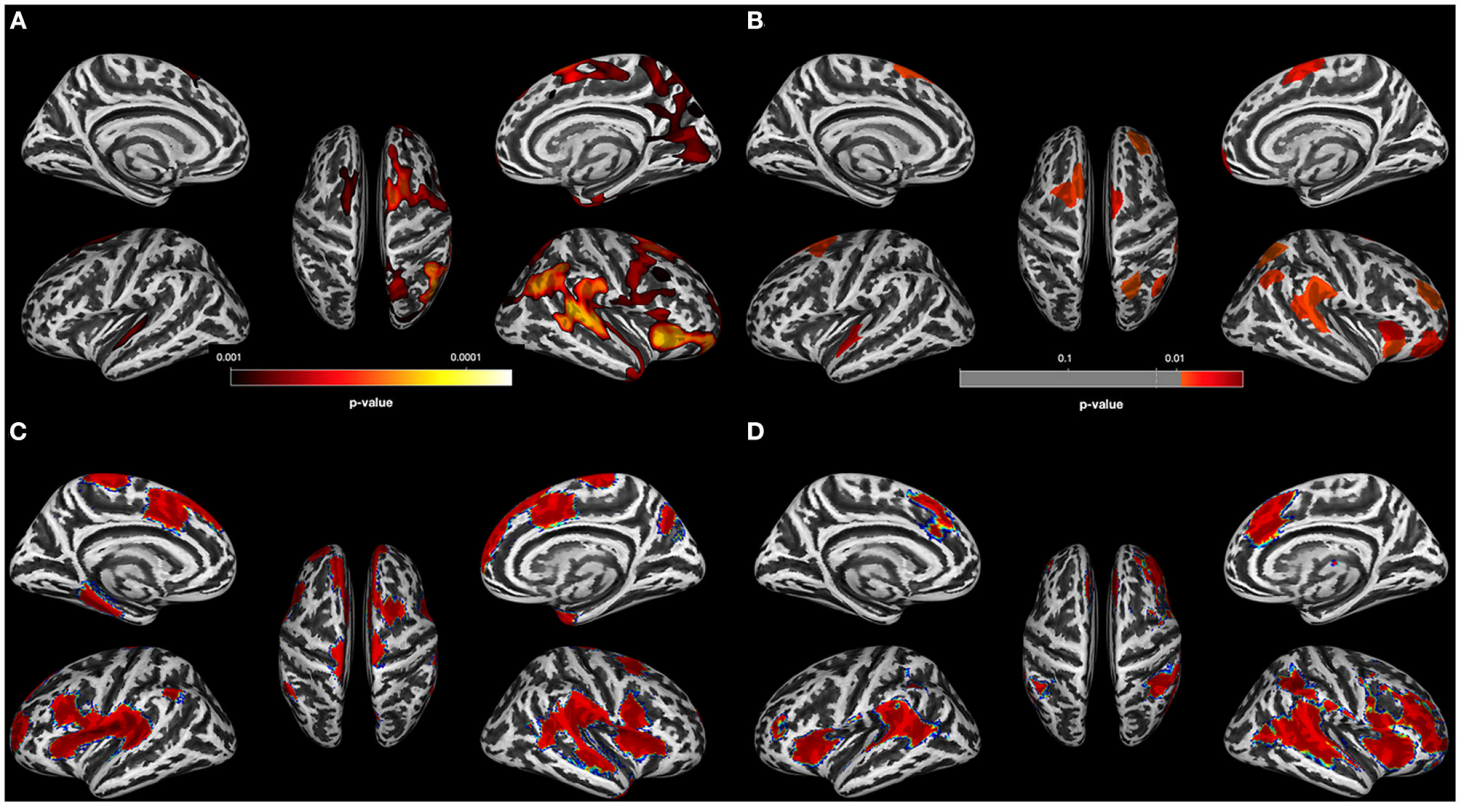
Results for OUD individuals. **(A)** Results of the data-driven vertex-wise cortical thickness (CT) analysis—controls have significantly higher CT values than opioid use disorder individuals (OUD) at *p* = 0.001 FWE corr. (TFCE). **(B)** Results of the data-driven ROI analysis with the Schaefer Atlas (48) (200 parcels) colored are ROIs for which controls have significantly higher mean CT values than OUD at *p* = 0.01 FDR corr; **(C)** CT covariance results, highlighted with red are all Schaefer ROIs with which the two ROIs corresponding to the two peak vertices of the most significant clusters in **(A)** show significant positive correlations at *p* < 0.05. **(D)** Result of the Neurosynth seed correlation analysis (thresholded at *r* > 0.2) with resting-state data of 1,000 healthy participants with the MNI coordinates of the two peak vertices of the most significant clusters in **(A)** as seeds.

The CT reduction pattern of the OUD individuals resembled the pattern found in the AUD group insofar as the OUD group also showed a more pronounced thinning in the right hemisphere. However, the spatial distribution of the clusters with CT reduction relative to controls was distinctly different between the two SUD subgroups (compare Figures 1A, 2A). The four right-hemispheric clusters in the OUD were located in the posterior part of the superior temporal gyrus extending into the temporoparietal junction and parietal cortex (29,779 vertices), in the insula extending into the inferior frontal gyrus (7,247 vertices), in the supplementary motor area extending into the motor cortex (9,850 vertices) and in the inferior frontal gyrus (552 vertices). Smaller clusters of thinning were found in the left temporal gyrus (1,498 vertices) and left superior frontal gyrus (1,751 vertices).

**Figure 3:**
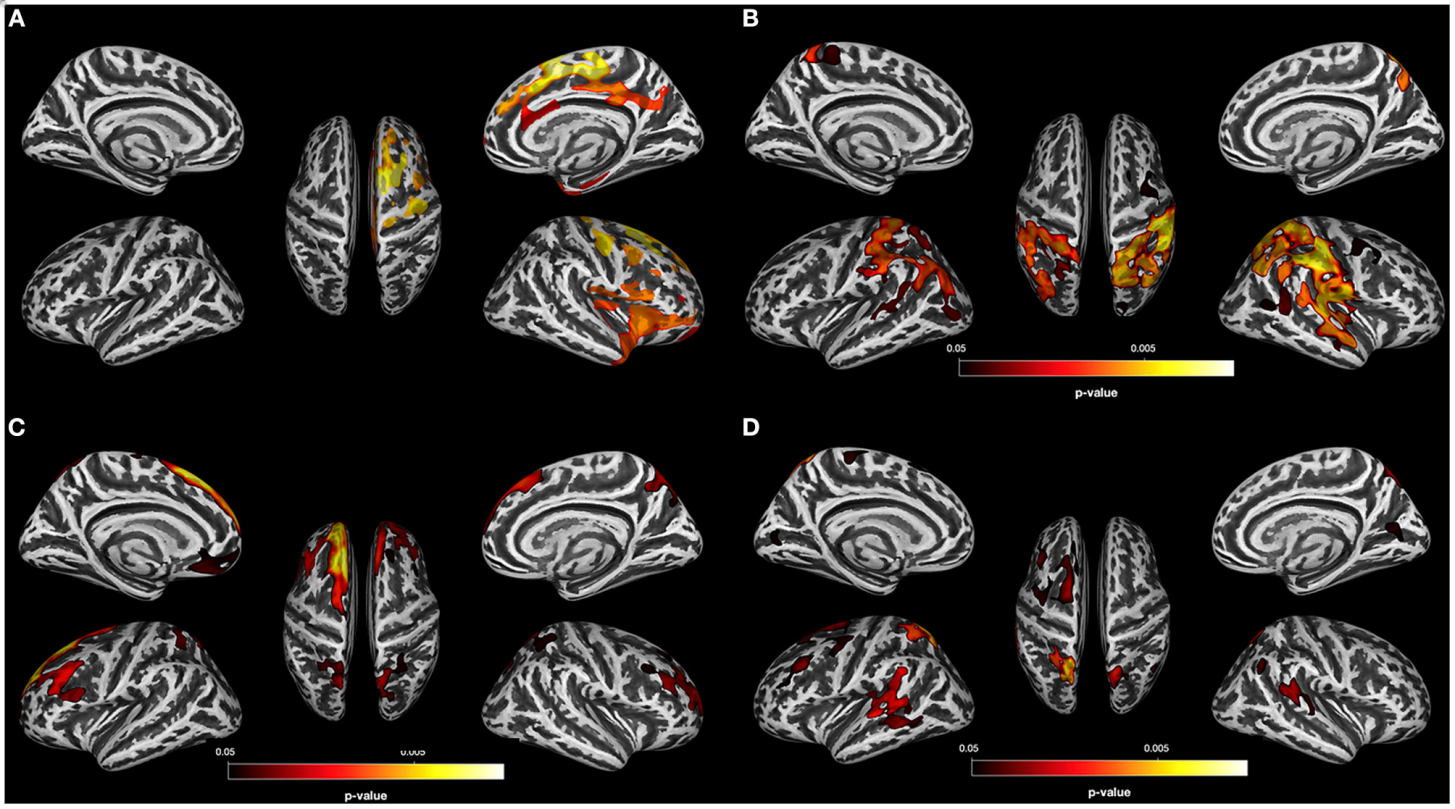
Results for PSU individuals. **(A)** Result of the data-driven vertex-wise cortical thickness (CT) analysis—controls have significantly higher CT values than polysubstance use disorder individuals (PSU) at *p* = 0.05 uncorr. (TFCE). **(B)** Result of the vertex-wise regression analysis—CT clusters in PSU with significant positive correlations with the BIS 11 subscore “High Attentional Impulsivity” at *p* < 0.05 FWE corr. (TFCE). **(C)** Result of the data-driven vertex-wise CT analysis—PSU have significantly higher CT values than alcohol use disorder individuals (AUD) at *p* = 0.05 FWE corr. (TFCE). **(D)** Result of the data-driven vertex-wise CT analysis—PSU have significantly higher CT values than opioid use disorder individuals (OUD) at *p* = 0.05 FWE corr. (TFCE).

Of the three SUD subgroups, only the PSU group did not show any region with significant CT reduction relative to controls. Since the threshold *p* < 0.001 FWE corr (TFCE) is very conservative, we gradually lowered the threshold to see if we could detect any regions with CT reduction in the PSU group relative to controls. Only when using a very lenient statistical threshold (*p* = 0.05 uncorr), did the PSU begin to show minimal cortical thinning concentrated in the right anterior part of the brain relative to controls (see Figure 3A for the location of these non-significant clusters with thinner cortex).

#### Vertex-Wise CT Comparisons Among the Three SUD Subgroups

Next, we compared the three SUD subgroups with each other at *p* = 0.001 FWE corr. (TFCE). Only the comparisons of the AUD and OUD groups with the PSU participants showed significant CT differences: Compared to PSU, AUD had lower CT values in the left superior frontal gyrus and posterior part of the middle frontal gyrus as well as in the bilateral precuneus (Figure 3C), whereas OUD had thinning in the left posterior part of superior frontal gyrus and the bilateral temporo-parietal junction (Figure 3D).

#### Groupwise Cerebellar and Subcortical GM Volume Comparisons

PSU and OUD subgroups did not show any voxel-wise subcortical or cerebellar GM volume losses relative to controls, neither at strict [*p* = 0.001 FWE corr. (TFCE)] nor more lenient statistical thresholds (*p* = 0.05 uncorr.). In contrast, AUDs showed extensive GM volume reductions relative to controls [*p* < 0.001 FWE corr. (TFCE)] in five clusters. The largest cluster (716 voxels) was located in the right cerebellar lobe VI, followed by clusters in the left cerebellar lobe VIIb (434 voxels), in the right thalamus extending into the right hippocampus (230 voxels), and additional clusters in the cerebellum (right cerebellar lobe VIIa (128 voxels) and left cerebellar lobe VI (61 voxels). Among the SUD subgroups, the AUD group showed significantly less GM than PSU participants in the cerebellum in one large cluster (5,695 voxels) in the left cerebellar lobes V–VI as well as significantly less GM than OUD individuals in an even more extensive cluster (11,135 voxels) in the left vermis 8 extending bilaterally into the corresponding cerebellar lobes. PSU and OUD individuals did not differ from each other in subcortical and cerebellar volumes, even after using a more lenient statistical threshold of *p* = 0.001 uncorr.

#### *Post*-*hoc* Analysis: Correlation of Self-Reported Impulsivity With Vertex-Wise CT

The goal of the *post-hoc* analysis was to put into context two of the PSU-specific findings. Despite having a lifetime history of alcohol consumption similar to that in AUD and as many years of chronic substance use as the OUD individuals, PSU individuals did not reveal significant cortical thinning or subcortical-cerebellar volume loss relative to controls; however, PSU patients consistently had the highest scores of all four groups in self-reported impulsiveness (see Table 1). To test the possibility that the high impulsivity scores in the PSU group could have a GM correlate, in particular subtle regional cortical thickening, we computed within the PSU group four *post-hoc* vertex-wise regression analyses (TFCE; non-parametric permutation with 10,000 iterations; threshold *p* = 0.05 FWE corrected) with all BIS scores separately and with age as a covariate without interest. We found that the attentional impulsivity subscore was positively associated with the CT values in five clusters predominantly located in the posterior part of the brain. The most extensive clusters were located in the right postcentral gyrus (30,856 vertices), the left superior parietal lobe (17,677 vertices), and the right middle frontal gyrus (1,258 vertices); smaller clusters were observed in the right cuneus (534 vertices) and right superior frontal gyrus (119 vertices; see Figure 3B). Similar exploratory regression analyses with the BIS 11 scores for the other SUD groups or controls did not yield any significant associations with regional CT values.

### Second Part—Mapping the Group-Specific Differences in CT to System-Level Functional Processes

As the PSU did not differ significantly from controls in regional CT, and our planned follow-up analyses required the coordinates of the peak vertices of the two clusters with the most significant cortical thinning as input, we only report the following results for the AUD and OUD groups.

**Figure 4:**
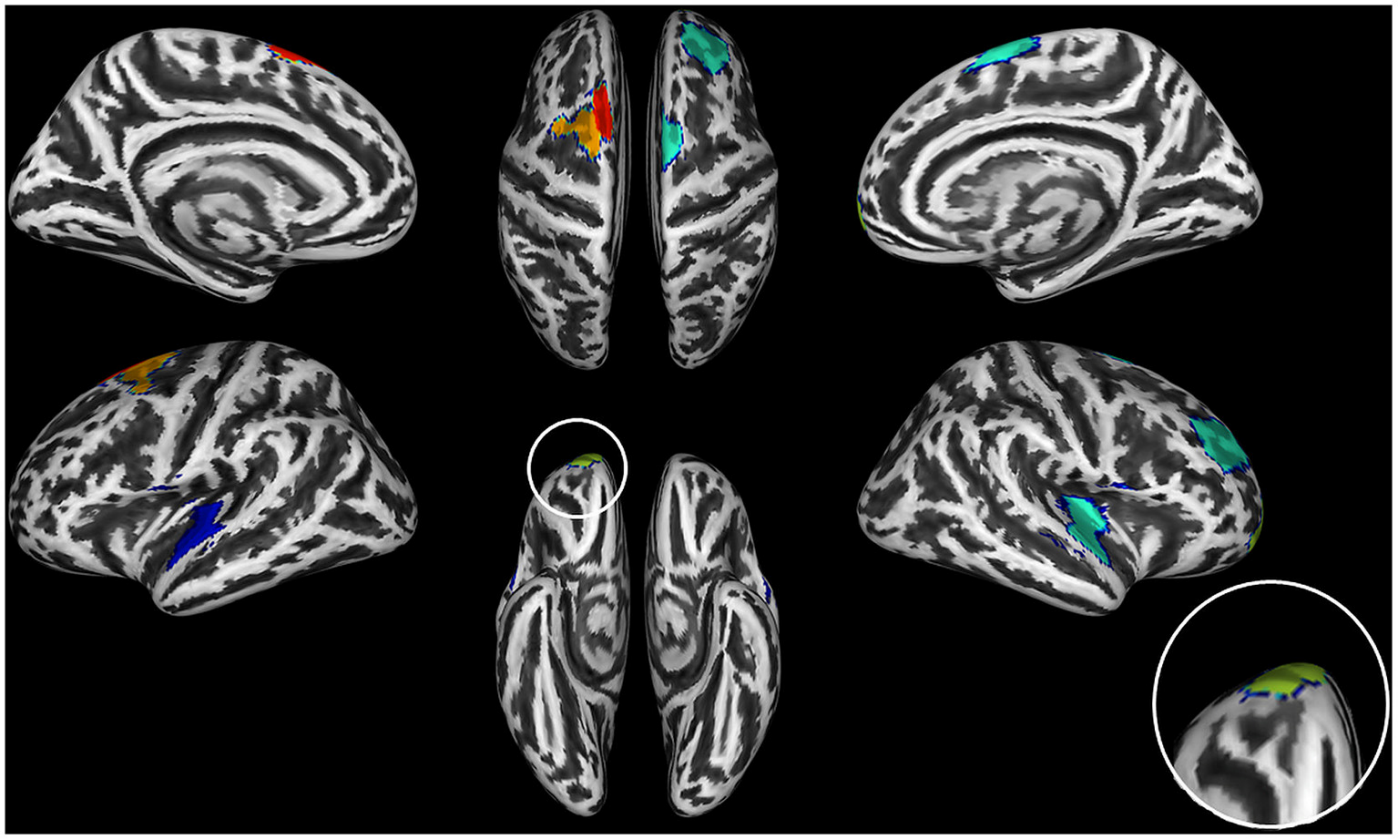
Brain regions with significant thinning in both AUD and OUD individuals when compared to controls. The seven shared brain ROIs showing significantly reduced cortical thickness in both alcohol use disorder (AUD) and opioid use disorder (OUD) individuals when compared with controls. Dark blue, Somatosensory Network; green, Salience/VentralAttention Network; light green, Limbic Network; orange, Executive Control Network; red, Default Mode Network. The inset on the right side of the figure shows an increase of the orbitofrontal cortex ROI, the region with the most significant thinning in all three substance use disorder (SUD) subgroups.

The data-driven ROI analyses with the Schaefer parcellation—to match the vertex-wise regional CT reduction pattern in the AUD and OUD individuals to 17 well-established ICNs—revealed that the thinner cortical regions in AUD and OUD individuals clearly differed in their network affiliation (Table 3 lists the results in detail). The 35 Schaefer parcels with significant CT reduction in the AUD group mainly belonged to three ICNs: the DMN (nine parcels), the ECN (eight parcels) and the combined SAL/VAN network (six parcels, Figure 1B). Interestingly, there was a hemispheric asymmetry with six of the 10 left-sided parcels with reduced CT belonging to the DMN and 15 right-sided parcels belonging predominantly to the ECN (six parcels) and SAL/VAN (five parcels) networks. The 14 Schaefer parcels with significant CT reduction in OUD, however, showed a more diverse ICN assignment (Figure 2B), with most of the parcels belonging to the right-sided SAL/VAN regions, while the rest were located in parcels belonging to the ECN, the temporoparietal network, the DAN, and the SMN. Only five Schaefer parcels with thinner cortices were shared by both AUD and OUD individuals: three of them in the right-sided SAL/VAN network, one in the left-sided DMN and one in left-sided SMN (Figure 4).

**Table 3:** Common and substance use-specific brain regions with CT reduction.

**Group**	**Intrinsic connectivity network**	**Hemisphere**	**ROI name according to Schaefer et al. (48)**
Common in AUD and OUD	Default mode network	L	DMNb_PrefrontalCortex_dorsal_4
	Executive control network	L	ECNa_PrefrontalCortex_dorsal_1
	Attention networks	R	SAL/VANa_FrontalMedial_2
			SAL/VANb_PrefrontalCortex_lateral_1
			SAL/VANb_Insula_2
	Limbic network	R	Limbicb_OrbitofrontalCortex_4
	Somatomotor network	L	SMNb_Aud_1
Exclusive for AUD	Default mode network	L	DMNa_PrefrontalCortex_medial_2
			DMNa_PrefrontalCortex_dorsal_2
			DMNb_PrefrontalCortex_dorsal_3
			DMNb_PrefrontalCortex_dorsal_2
			DMNb_PrefrontalCortex_dorsal_1
		R	DMNa_PrefontalCortex_medial_3
			DMNa_PrefontalCortex_dorsal_1
			DMNb_PrefontalCortex_dorsal_1
	Executive control network	L	ECNa_PrefrontalCortex_dorsal_1
			ECNb_PrefrontalCortex_lateral_ventral_2
		R	ECNa_PrefrontalCortex_dorsal_1
			ECNa_PrefrontalCortex_lateral_1
			ECNb_PrefrontalCortex_medial_posterior_1
			ECNb_PrefrontalCortex_lateral_dorsal_3
			ECNb_PrefrontalCortex_lateral_ventral_2
			ECNb_PrefrontalCortex_lateral_dorsal_2
	Attention networks	L	SAL/VANb_PrefrontalCortex_lateral_1
		R	SAL/VANa_PrefrontalCortex_medial_1
			SAL/VANb_PrefrontalCortex_lateral_ventral_1
Exclusive for OUD	Executive control network	R	ECNb_PrefrontalCortex_lateral_ventral_1
			ECNb_IntraparietalLobe_1
	Attention networks	R	SAL/VANa_ParsOpercularis_1
			SAL/VANb_Insula_1
			SAL/VANb_PrefrontalCortex_lateral_1
			DANa_SuperiorParietalLobe_4
	Temporoparietal network	R	Temporparietal_Region_4
	Somatomotor network	R	SMNb_Auditory_2

*The table lists the results of the data-driven ROI analysis at p < 0.01 FDR corr.; AUD, alcohol use disorder individuals; OUD, opioid use disorder individuals. The naming of the regions of interest (ROI) follows the convention of the Schaefer parcellation (48), which assigns the 200 ROI to 17 different networks with corresponding subnetworks differentiated by lower case letters: DMN, default mode network; ECN, executive control network; SAL, salience network; DAN, dorsal attention network; VAN, ventral attention network; SMN, somatomotor network*.

Figure 1C for the AUD and Figure 2C for the OUD individuals show the structural covariance maps of all Schaefer ROIs with which the two “seed ROIs” had a significant positive correlation. Figure 1D shows the combined results of the SCAs with the MNI coordinates of the two peak vertices of the most significant clusters from the comparison AUD individuals vs. controls thresholded at *r* > 0.2, Figure 2D shows the results of the corresponding analyses in the OUD group. In the AUD group, the two seed vertices were in the right and left superior frontal gyrus, which both functionally belong to the DMN. Accordingly, the Neurosynth SCA results for the AUD group's two peak voxels (Figure 1D) corresponded to the spatial distribution of the DMN (51) and covered the bilateral medial and lateral parts of the superior frontal gyrus, posterior middle frontal gyrus, part of the lateral inferior frontal gyrus, middle temporal gyrus, angular gyrus, and the bilateral crus of the cerebellum. The OUD group's two seed vertices were located in the posterior part of the superior temporal gyrus near the border to the parietal lobe and in the right anterior insula. Consequently, the Neurosynth SCA revealed a bilateral network resembling an enlarged SAL with the two SAL core regions, bilateral insula, and anterior cingulate gyrus, but also encompassing the bilateral temporo-parietal junction and middle frontal gyrus (Figure 2D). To quantify the similarity of the structural covariance maps with their corresponding SCA map, we then computed the percentage of the voxels shared between the two maps, the number of voxels of the SCA maps were used to define the 100% reference, since they covered the smaller number of voxels in both AUD and OUD. In the AUD group, 62.6% of the voxels of the functional SCA map were shared with the structural covariance map, which is in the same range as the structure-function concordance of 64–68% reported earlier Luo et al. (25) and Vanasse et al. (26); it therefore allows the conclusion that the cortical reduction pattern in the AUD group recapitulated the DMN on a system-level. In the OUD group, only 36.2% of the voxels were shared between the functional SCA map and the structural covariance map. This comparatively low concordance should be evaluated in the following context: one, the two seed ROIs/seed voxels in OUD originated from two different networks (SAL and temporoparietal network) in contrast to the two seeds in the AUD that both were located in the DMN, and, two, SAL is defined by the Schaefer parcellation as a combination of salience and ventral attention networks.

## Discussion

Chronic SUD is associated with brain-wide GM alterations, but most dominantly in frontal regions (2). One aim of our study was to investigate common and substance-specific GM alterations in three different SUD subgroups (AUD, PSU, and OUD) in comparison to healthy controls and to each other. A second aim was to better understand the GM reduction patterns found in the three SUD subgroups on a more system-level way by relating them to functional brain systems operationalized as ICNs and their corresponding CT covariance networks.

We found that both AUD and OUD participants showed significant CT reductions relative to controls, particularly in right frontal brain regions, whereas PSU individuals did not. Furthermore, only AUD had significantly smaller subcortical structures (thalamus and hippocampus) and cerebellum relative to controls, OUD and PSU individuals. The latter two did not show significant subcortical or cerebellar GM volume differences compared to controls or with each other. Except for seven shared ROIs (see Figure 4, Table 3), six of them in the frontal cortex, the CT, and GM volume atrophy patterns in all three SUD subgroups showed distinctly different spatial distributions, which indicates that different and substance-specific functional systems were affected by AUD and OUD. In the following paragraphs, we discuss these findings in more detail.

### Common CT Features in All Three SUD Subgroups

AUD and OUD participants, as well as PSU individuals before correction for multiple comparisons, shared a common set of three brain regions with cortical thinning in the posterior part of the right medial superior frontal gyrus, the right insula and the right orbitofrontal cortex. The latter showed the highest degree of CT reduction among the three common regions. The orbitofrontal cortex, especially its medial part, is functionally connected with the nucleus accumbens and belongs to the core regions of the brain reward system (52). The two other brain regions with common thinning belong functionally belong to the SAL. These findings are consistent with those of Mackey et al. (2), who also identified orbitofrontal cortex and insula as commonly thinner in individuals with alcohol, cocaine, methamphetamine, or nicotine use disorder. Further, our results complement the findings of the mega-analysis by Mackey et al. (2) by showing that both OUD individuals and PSU individuals (but only before correction for multiple comparisons) share this substance disorder-related GM signature. In addition, OUD and AUD participants shared another four common regions with thinning in bilateral frontal regions and in the right superior temporal gyrus (Figure 4) functionally belonging to the DMN, ECN, and temporoparietal network. The three SUD subgroups had two other features in common: First, all three subgroups showed asymmetric CT reduction to varying degrees, but with more pronounced thinning in the right hemisphere; second, in all three SUD subgroups the CT reduction was not constrained to just one ICN but they all showed GM alterations in multiple ICNs, most pronounced in the SAL, the DMN, and the ECN, where the data-driven *post-hoc* ROI analyses had identified the highest number of ROIs with significant CT reductions (see Table 3).

### Substance-Specific Findings: AUD

The AUD group had two distinct GM features in which they differed from the two other SUD subgroups. First, they showed the quantitatively most serious detrimental effects of substance disorder of the three SUD subgroups studied. They not only showed the spatially most extended CT reduction pattern relative to controls (72,274 vertices compared to 49,034 vertices in OUD), they also had significantly smaller global WM volume than controls and the other two SUD subgroups, and as a consequence also significantly higher global brain atrophy than OUD and PSU groups. In that aspect our findings are consistent with the findings of Mackey et al. (2), who also found that AUD individuals showed the spatially most serious CT alterations of all five SUD subgroups investigated (cocaine, methamphetamine, nicotine, and cannabis). Second, the AUD individuals were the only SUD subgroup with significant cerebellar and subcortical GM volume loss relative to controls and the other SUD subgroups. Taken together, the CT reduction pattern and the subcortical-cerebellar GM volume losses point to the frontocerebellar circuit as the brain system with the most extensive GM alterations in AUD. That the frontocerebellar circuit is especially affected in AUD patients is not new (53–57). However, the finding that extensive GM loss in the frontocerebellar circuit seems a unique GM signature for “pure” AUD patients is new and remarkable, because such GM loss appears non-detectable in age- and education-matched PSU individuals with comparable lifetime alcohol use and tobacco use histories.

The frontocerebellar circuit can be further divided into functionally distinct subcircuits corresponding to well-known ICNs such as the DMN, ECN, SAL, DAN, and SMN subcircuits (58–60). The assignment of the brain regions with CT reduction to ICNs pointed to the DMN, the ECN, and the combined SAL/VAN as the three networks mainly affected by AUD. Of these ICNs, the CT covariance analysis as well as the Neurosynth SCA analysis (with the two peak vertices from the CT analysis as seed) highlighted the DMN as being particularly relevant for the understanding of AUD, because both the resulting CT covariance pattern and the resting-state connectivity pattern closely matched the regions forming the DMN (51). The importance of the DMN for understanding AUD-related brain alterations is further supported by the fact that several of the core regions of the brain reward system such as ventral tegmental area, nucleus accumbens and caudate nucleus are functionally strongly connected with the DMN (51, 61) and there even is evidence that the DMN can exert a top-down influence on the ventral tegmental area (62).

At a system-level, the fine-tuned interplay between SAL, ECN, and DMN is necessary for the brain to switch from a state of quiet awake rest with high activity of the DMN to active goal-driven behavior and cognition, which demands high activity in the task-positive networks such as the ECN and the attention networks. The SAL, in particular the right anterior insula, has been shown to initiate that switching (63–65). A failure to suppress DMN activity is associated with severe cognitive deficits (66). The interaction between DMN, SAL and ECN is mediated by dopamine (67–71) as well as glutamate, and gamma-aminobutyric acid (GABA) (72–74), and all three neurotransmitters have been shown to be imbalanced in AUD (52, 75–78).

### Substance-Specific Findings: OUD

The OUD group also had two distinct GM features in which they differed from the other two SUD groups. First, the OUD group had significantly lower average CT values than controls, AUD and PSU individuals. Second and probably related to that finding, the OUD group had two hotspots with significant cortical thinning (right posterior superior temporal gyrus and the right anterior insula) belonging to two different ICNs. Cortical thinning in these two regions is in line with previous reports on OUD: in a meta-analysis of 12 VBM studies in OUD Wollman et al. (79) identified four predominantly right-sided fronto-temporo regions, the right Heschl's gyrus in the posterior superior temporal gyrus, the right middle frontal gyrus, the right gyrus rectus, and the left temporal pole GM, as the primary sites with GM loss in OUD. Bach et al. (80) and Bach et al. (81) found in two separate data-driven VBM analyses using CAT12 in OUD individuals on maintenance therapy less GM in the bilateral insula among other brain regions. The volume reduction in the right insula was associated with higher social rejection sensitivity (80) and with more errors during a working memory task (81).

Also in the OUD group, regions with CT reductions belong to different ICNs, primarily the SAL and to a lesser degree the DMN, ECN, SMN, and the limbic and temporoparietal networks. The structural covariance analysis and the Neurosynth SCA for the OUD highlighted the SAL and the SMN as system-level relevant. The particular importance of these two ICNs in OUD is consistent with the findings of Khalili-Mahani et al. (82), who found that these ICNs showed the most extensive functional effects to acute opioid intake in healthy volunteers. Furthermore, Galaj and Xi (83) recently postulated that cocaine and heroin are mediated by different mechanisms in the brain insofar as dopamine release from both the ventral tegmental area and the substantia nigra is equally rewarding for opioids (but not cocaine). The additional involvement of the substantia nigra in OUD could explain why our system-level analyses highlighted the importance of these two ICNs, because the nigrostriatal pathway projects from the substantia nigra primarily to the dorsal striatum and the SMN (69). Shafei et al. (84), using a pharmacological intervention in healthy participants, showed that the interaction of both the SMN and SAL with the other major ICNs of the brain is modulated by dopamine. A transient dopamine depletion led to a significant increase of the BOLD signal variability exclusively in SMN and SAL. Furthermore, the authors found that these two ICNs together with the temporoparietal network also were not any longer able to synchronize with other ICNs during the transient dopamine depletion condition (84).

### Substance-Specific Findings: PSU

The PSU group had three GM features that set it apart from the other SUD groups. First, in contrast to the AUD and OUD groups, PSU individuals had no cortical thinning compared to controls using whole-brain vertex-wise analyses at conventional statistical thresholds (thinning is seen at lower statistical thresholds in right anterior brain). This seems at odds with earlier results of group comparisons by VBM (10) and Freesurfer (6), that described reduced GM in several frontal brain regions and temporal and precentral gyri compared to controls. In these two reports, PSU individuals also showed less GM than “pure” AUD patients: in subcortical brain and brainstem (10) as well as thinning in the anterior cingulate gyrus (6). It is difficult to directly compare our vertex-wise CT results with VBM results of voxel-wise GM concentration, as the degree to which the cortical thickness, cortical surface, and volume are related to each other is still under debate (85, 86). The argument of difficult comparability also applies to the study of Pennington et al. (6). Methodical and technical differences between these studies such as magnetic field strengths used, a priori selected ROI vs. data-driven vertex-wise CT analysis, different CT computations by Freesurfer and CAT12 (39, 87–89), using TIV as a covariate without interest or not (40–42), all these differences might contribute to study differences, apart from biological variation contributed by the specific populations enrolled for study. Nevertheless, the incongruent GM results of the three studies could also be an indication that the effects of PSU on the brain must be understood as complex interactions between neural processes that lead to measurable changes in WM tissue (12) as well as alterations in cortical regions that might mask frank volume loss detectable by gross structural MRI (90).

Another feature that set the PSU group apart from the other SUD groups is relatively high self-reported impulsivity and its cortical structural correlate. Only in the PSU group was the BIS 11 sub-score “attentional impulsivity” (defined as an inability to focus attention or concentrate) positively correlated with cortical thickness in the bilateral superior parietal cortex. This part of the brain is largely identical to the posterior parts of the DAN [intraparietal sulcus, superior parietal lobe and dorsal frontal cortex along the precentral sulcus and frontal eye fields; (91, 92)] and the VAN [temporoparietal sulcus, frontal operculum, and anterior insula; (91, 92)]. The DAN and VAN are functionally independent networks that are both involved in redirecting attention. In particular, the DAN is top-down engaged in focused goal-driven attention (91, 93, 94), during which the VAN is usually suppressed by the DAN but gets activated in response to unexpected but behaviorally relevant stimuli (91, 94). However, the positive correlation of the BIS 11 sub-score “attentional impulsivity” with the CT values of predominantly inferior and superior parietal regions in the PSU participants should not be interpreted as an indicator of SUD-related thickening in these regions, because the same association between “attentional impulsivity” and these parietal regions was also found in a VBM study with healthy controls (95).

The third PSU-unique GM feature were regions with thicker cortices than in the AUD and OUD groups. Remarkably, those regions were identical to the regions with cortical thinning in AUD vs. controls (left DMN regions in the superior frontal gyrus) or OUD vs. controls (right posterior part of the superior temporal gyrus). These findings of relatively preserved cortical thickness in PSU with extensive lifetime substance use histories further highlight the substance-related distinctiveness of the CT reduction patterns in AUD and OUD.

### Limitations

A limitation of our study is certainly the unbalanced sample sizes with 65 AUD individuals in relation to 21 PSU, 27 OUD individuals, and 21 controls. Therefore, we cannot exclude the possibility that we might have been able to detect significant CT differences between the PSU group and controls with larger sample sizes. The concern that the PSU group was too small to reveal significant group differences is mitigated by the fact that we were able to observe significant differences between PSU and OUD individuals, although the latter group had only six participants more than the PSU group. Further, our finding that the AUD group had much more serious cortical thinning than the other two SUD subgroups when compared to controls could have been driven by the fact that we had 2–3 times more AUD than OUD and PSU participants. To rule out that possibility, we repeated our vertex-wise CT analysis with an AUD sample of the same size as the other two SUD subgroups and controls: This smaller group still showed a much more extensive CT reduction than the OUD and the same characteristic GM alteration pattern with the DMN frontocerebellar subcircuit as the most affected system. Finally, one of the main results of our study was that AUD and OUD cannot simply be described as a dysfunction of a single network, but that the interactions of several networks seem to be disturbed in AUD as well as OUD. To relate our findings in a meaningful way to clinical treatment and treatment outcome, functional fMRI data, and appropriate advanced network analyses in combination with cognitive/behavioral data are needed to disentangle the dysfunctional interactions of the involved networks and their potential clinical ramifications.

## Conclusions

Using vertex-wise data-driven CT and VBM analyses in combination with *post-hoc* data-driven ROI analyses, we identified both common cortical thinning in right anterior brain as well as SUD-specific regional GM alterations among AUD and OUD. Furthermore, the similarity between the *post-hoc* computed SCA connectivity and CT covariance patterns suggests that processes at the system-level of the intrinsic brain architecture underlie the cortical thinning prevalent in AUD and OUD individuals. As the intrinsic brain architecture and the interaction between the different ICNs is partly modulated by various neurotransmitter systems that are known to be out-of-balance in SUDs, future studies might combine system-level approaches such as ICNs and GM covariance analyses with methods able to quantify neurotransmitters or receptor density across these systems. Such multimodal analyses might be critical to better understand the unique ways different SUDs affect the brain and to inform treatments for specific SUDs.

## Data Availability Statement

Restrictions apply to the datasets: the datasets for this article are not publicly available, because they were created as part of Veteran's Administration approved research. Requests to access the datasets should be directed to the PI of the study David L. Pennington (david.pennington2@va.gov).

## Ethics Statement

The studies involving human participants were reviewed and approved by Committee on Human Research at the University of California San Francisco Committee on Human Research and the San Francisco VA Health Care System.

## Author Contributions

DM: conceptualization, funding acquisition, resources, supervision, and writing—review and editing. DP: conceptualization, supervision, and writing—review and editing. AM: conceptualization, formal analysis, methodology, visualization, and writing—original draft preparation, reviewing, and editing. All authors contributed to the article and approved the submitted version.

## Funding

This work was supported by NIH AA010788 (DM), DA039903 (DM then DP), DoD W81XWH-15-2-0020 (DM), Department of Veterans Affairs, Career Development Award–2 1IK2CX001510-01 (DP), and by San Francisco VA Health Care System resources. The research was administered by the Northern California Institute for Research and Education. The funding and administrative agencies had no role in the design of the study, the collection, and analysis of data or the decision to publish.

## Conflict of Interest

The authors declare that the research was conducted in the absence of any commercial or financial relationships that could be construed as a potential conflict of interest.

## Publisher's Note

All claims expressed in this article are solely those of the authors and do not necessarily represent those of their affiliated organizations, or those of the publisher, the editors and the reviewers. Any product that may be evaluated in this article, or claim that may be made by its manufacturer, is not guaranteed or endorsed by the publisher.
